# Supplementary material for: Discovery of a novel coltivirus in a newly identified Bat Bug Species (Heteroptera: Cimicidae) in Cambodia
Source: PLoS Negl Trop Dis. 2026 Jun 8;20(6):e0014372. doi: 10.1371/journal.pntd.0014372 (PMC13258151; doi:10.1371/journal.pntd.0014372)
Supplement: S1 Table — (DOCX) [file pntd.0014372.s001.docx]

**S1 Table: COI, 16S and 18S Sequences of Cimicidae species and outgroups obtained from NCBI GenBank.**

| **Species** | **Host group** | **Host species** | **Country** | **COI** | **16S** | **18S** |
| --- | --- | --- | --- | --- | --- | --- |
| Cimicid 019 | Bat | *Mops plicatus* | Cambodia | PV273274 | PV275595 | PV275585 |
| Cimicid 049 | Bat | *Mops plicatus* | Cambodia | PV273269 | PV275596 | PV275586 |
| Cimicid 060 | Bat | *Mops plicatus* | Cambodia | PV273275 | PV275597 | PV275587 |
| Cimicid 070 | Bat | *Mops plicatus* | Cambodia | PV273276 | PV275598 | PV275588 |
| Cimicid 076 | Bat | *Mops plicatus* | Cambodia | PV273272 | PV275599 | PV275589 |
| Cimicid 077 | Bat | *Mops plicatus* | Cambodia | PV273273 | PV275600 | PV275590 |
| Cimicid 083 | Bat | *Mops plicatus* | Cambodia | PV273268 | PV275601 | PV275591 |
| Cimicid 084 | Bat | *Mops plicatus* | Cambodia | PV273270 | PV275602 | PV275592 |
| Cimicid 087 | Bat | *Mops plicatus* | Cambodia | PV273271 | PV275603 | PV275593 |
| *Acanthocrios furnarii* | Bird | *Furnarius rufus* (nest) | Brazil | MG596830 | MG596866 | MG978385 |
| *Afrocimex constrictus* | Bat | *Rousettus aegyptiacus* | Kenya | MG596805 | MG596842 | MG978358 |
| *Afrocimex constrictus 2* | Bat | *Rousettus aegyptiacus* | Kenya | MG596806 | MG596843 | MG978359 |
| *Aphrania barys* | Bat | *Neoromicia capensis* (currently *Laephotis capensis*) | Namibia | MG596820 | MG596856 | MG978375 |
| *Aphrania barys 2* | Bat | *Neoromicia capensis* (currently *Laephotis capensis*) | South Africa | MG596825 | MG596861 | MG978380 |
| *Aphrania elongata* | - | Unknown | Senegal | MG596812 | MG596849 | MG978367 |
| *Aphrania elongata 2* | Bat | *Scotophilus leucogaster* | Mauritania | KF018763 | KF018729 | KF018715 |
| *Aphrania recta* | Bat | *Nycticeinops schlieffeni* | Mauritania | KF018764 | KF018730 | KF018716 |
| *Bucimex chilensis* | - | Unknown | Chile | MG596840 | MG596877 | MG978399 |
| Cacodminae gen. sp. | Bat | *Scotophilus leucogaster* | Mauritania | KF018764 | KF018730 | KF018716 |
| *Cacodmus sparsilis* | Bat | *Pipistrellus dhofarensis* | Oman | MG596813 | MG596850 | MG978369 |
| *Cacodmus vicinus* | Bat | *Scotoecus hirundo* | Senegal | MG596819 | MG596855 | MG978374 |
| *Cacodmus vicinus* 2 | Bat | *Pipistrellus* sp. | Spain | MG596816 | MG596852 | MG978371 |
| *Cacodmus villosus* | Bat | *Pipistrellus hesperidus* | Ethiophia | MG596821 | MG596857 | MG978376 |
| *Cacodmus villosus* 2 | Bat | *Neoromicia capensis* (currently *Laephotis capensis*) | Namibia | MG596823 | MG596859 | MG978378 |
| *Cimex adjunctus* | Bat | *Nycticeius humeralis* | USA | GU985536 | GU985558 | KF018712 |
| *Cimex emarginatus* | Bat | *Myotis* cf. *alcathoe* | Bulgaria | MG596837 | MG596874 | MG978396 |
| *Cimex emarginatus* 2 | Bat | *Pipistrellus pipistrellus* | Morocco | MF680526 | MF680517 | MG978397 |
| *Cimex hemipterus* | Human | *Homo sapiens* | Kenya | MG596826 | MG596862 | MG978381 |
| *Cimex hemipterus 2* | Human | *Homo sapiens* | Malaysia | KF018754 | KF018724 | KF018710 |
| *Cimex latipennis* | Bat | *Myotis lucifugus* | Canada | KF018758 | KF018734 | KF018720 |
| *Cimex latipennis 2* | Bat | *Myotis volans* | Canada | KF018757 | KF018733 | KF018719 |
| *Cimex lectularius* | Human | *Homo sapiens* | Czechia | GU985524 | GU985546 | KF018711 |
| *Cimex lectularius 2* | Human | *Homo sapiens* | UK | MG596836 | MG596873 | MG978394 |
| *Cimex pipistrelli* | Bat | *Pipistrellus* sp. | UK | GU985534 | GU985556 | MG978393 |
| *Cyanolicimex patagonicus* | Bird | *Cyanoliseus patagonus* | Argentina | MG596833 | MG596869 | MG978388 |
| *Haematosiphon inodorus* | Bird | *Falco mexicanus* (nest) | USA | MG596829 | MG596865 | MG978384 |
| *Latrocimex spectans* | Bat | *Noctilio leporinus* | Belize | MW269881 | MW270938 | MZ378786 |
| *Leptocimex duplicatus* | - | Unknown | Israel | MG596810 | MG596847 | MG978365 |
| *Leptocimex inordinatus* | Bat | Chiroptera | Thailand | KT380161 | KT592538 | - |
| *Ornithocoris pallidus* | Bird | *Delichon urbicum* (nest) | USA | MG596828 | MG596864 | MG978383 |
| *Paracimex avium* | Bird | *Aerodramus salanganus* | Indonesia | MG596807 | MG596844 | MG978360 |
| *Paracimex chaeturus* | Bird | *Aerodramus brevirostris* | China | MF680531 | MF680520 | MG978362 |
| *Paracimex setosus* | Bird | *Aerodromus* sp. | Malaysia | KF018761 | KF018735 | KF018721 |
| *Primicimex cavernis* | Bat | *Tadarida brasiliensis* | Mexico | MG596839 | MG596876 | MG978398 |
| *Psitticimex uritui* | Bird | *Myiopsitta monachus* | Argentina | MG596831 | MG596867 | MG978386 |
| *Stricticimex sp.* | Bat | *Nyctinomus thomasi* (currently *Tadarida aegyptiaca*) | Oman | MG596817 | MG596853 | MG978372 |
| *Stricticimex namru* | Bat | Chiroptera | Iran | MG596811 | MG596848 | MG978366 |
| *Amphiareus obscuriceps* | Outgroup |  |  | GQ292178 | GQ258358 | GQ258393 |
| *Anthocoris confusus* | Outgroup |  |  | KM022525 | GQ258359 | GQ258401 |
| *Capsus ater* | Outgroup |  |  | AY252977 | AY252712 | EU683117 |
| *Dysepicritus rufescens* | Outgroup |  |  | GQ292210 | GQ258386 | GQ258399 |
| *Eteoneus angulatus* | Outgroup |  |  | EF523481 | EF487290 | EF487311 |
| *Himacerus apterus* | Outgroup |  |  | KR034788 | GQ258381 | GQ258425 |
| *Lasiochilus japonicus* | Outgroup |  |  | GQ292187 | GQ258367 | GQ258410 |
| *Loricula elegantula* | Outgroup |  |  | KM022867 | EU683098 | EU683151 |
| *Lyctocoris beneficus* | Outgroup |  |  | GQ292284 | GQ258369 | GQ258412 |
| *Nabis stenoferus* | Outgroup |  |  | GQ292211 | GQ258379 | GQ258426 |
| *Nabicula flavomarginata* | Outgroup |  |  | KM022694 | GQ258380 | GQ258424 |
| *Orius minutus* | Outgroup |  |  | KR040183 | GQ258372 | GQ258417 |
| *Scoloposcelis albodecussata* | Outgroup |  |  | GQ292129 | GQ258376 | GQ258422 |
| *Xylocoris cerealis* | Outgroup |  |  | GQ292172 | GQ258384 | GQ258395 |
