# Supplementary material for: Discovery of a novel coltivirus in a newly identified Bat Bug Species (Heteroptera: Cimicidae) in Cambodia
Source: PLoS Negl Trop Dis. 2026 Jun 8;20(6):e0014372. doi: 10.1371/journal.pntd.0014372 (PMC13258151; doi:10.1371/journal.pntd.0014372)
Supplement: S2 Table — (DOCX) [file pntd.0014372.s002.docx]

**S2 Table: Body measurements of *Stricticimex phnomsampovensis* Suor & Maquart sp. Nov.**

|  |  | **Holotype Male** | **Allotype Female** | **Paratype 1**  **Female** | **Paratype 2**  **Male** | **Paratype 3**  **Female** | **Paratype 4**  **Female** | **Paratype 5**  **Female (juvenile)** |
| --- | --- | --- | --- | --- | --- | --- | --- | --- |
|  | Accession n° | Ento_006 | Ento_001 | Ento_002 | Ento_006 | Ento_003 | Ento_004 | Ento_005 |
| **Head** | Lenght | 0,44 | 0,55 | 0,48 | 0,51 | 0,45 | 0,47 | 0,53 |
|  | Width | 0,41 | 0,51 | 0,49 | 0,48 | 0,53 | 0,54 | 0,45 |
|  | Eye | 0,1 | 0,09 | 0,08 | 0,76 | 0,067 | 0,07 | 0,07 |
|  | Interocular | 0,4 | 0,45 | 0,45 | 0,43 | 0,45 | 0,46 | 0,41 |
| **Antenna** | I | 0,15 | 0,15 | 0,15 | 0,16 | 0,13 | 0,14 | 0,15 |
|  | II | 0,42 | 0,45 | 0,46 | 0,47 | 0,47 | 0,45 | 0,43 |
|  | III | 0,7 | 0,83 | 0,79 | 0,78 | 0,67 | 0,75 | 0,58 |
|  | IV | 0,43 | 0,57 | 0,37 | 0,51 | 0,52 | 0,42 | 0,39 |
|  | **Total** | **1,7** | **2** | **1,77** | **1,92** | **1,79** | **1,76** | **1,55** |
| **Rostrum** | I | 0,2 | 0,23 | 0,2 | 0,2 | 0,2 | 0,2 | 0,17 |
|  | II | 0,18 | 0,21 | 0,17 | 0,2 | 0,2 | 0,2 | 0,16 |
|  | III | 0,1 | 0,13 | 0,1 | 0,13 | 0,1 | 0,17 | 0,08 |
|  | **Total** | **0,48** | **0,57** | **0,47** | **0,53** | **0,5** | **0,57** | **0,41** |
| **Pronotum** | Lenght | 0,28 | 0,3 | 0,27 | 0,3 | 0,28 | 0,3 | 0,23 |
|  | Width | 0,6 | 0,68 | 0,65 | 0,66 | 0,68 | 0,67 | 0,61 |
|  | Setae 1 | 0,16 | 0,17 | 0,17 | 0,17 | 0,19 | 0,17 | 0,17 |
|  | Setae 2 | 0,18 | 0,18 | 0,21 | 0,2 | 0,2 | 0,2 | 0,16 |
| **Hemelytras** | Lenght | 0,37 | 0,38 | 0,38 | 0,37 | 0,38 | 0,37 | Na |
|  | Width | 0,19 | 0,20 | 0,18 | 0,20 | 0,19 | 0,18 | Na |
| **Hind leg** | Femur | 0,79 | 0,94 | 0,83 | 0,81 | 0,88 | 0,93 | 0,75 |
|  | Tibia | 1,6 | 1,86 | 1,64 | 1,63 | 1,73 | 1,89 | 1,53 |
|  | Width F | 0,18 | 0,18 | 0,17 | 0,18 | 0,17 | 0,17 | 0,17 |
