# Supplementary material for: Discovery of a novel coltivirus in a newly identified Bat Bug Species (Heteroptera: Cimicidae) in Cambodia
Source: PLoS Negl Trop Dis. 2026 Jun 8;20(6):e0014372. doi: 10.1371/journal.pntd.0014372 (PMC13258151; doi:10.1371/journal.pntd.0014372)
Supplement: S3 Table — (DOCX) [file pntd.0014372.s003.docx]

**S3 Table. GenBank accession numbers.**

| **RdRp Gene** | |
| --- | --- |
| **Accession** | **Organism_Name** |
| AAZ94069 | Aedes pseudoscutellaris reovirus |
| AAL31497 | Chum salmon reovirus CS |
| AAM92745 | Golden shiner reovirus |
| ADZ31977 | Scophthalmus maximus reovirus |
| ABV01040 | American grass carp reovirus |
| WPR16556 | Avian associated coltivirus |
| UPN67769 | California hare coltivirus |
| WAK76799 | Colorado tick fever virus |
| AAM18357 | Colorado tick fever virus |
| WHS68118 | Colorado tick fever virus |
| UVC65861 | Colorado tick fever virus |
| NP_690891 | Colorado tick fever virus |
| UVC65874 | Colorado tick fever virus |
| XCO66239 | Colorado tick fever virus |
| AAK73521 | Lymantria dispar cypovirus 1 |
| AAK73087 | Lymantria dispar cypovirus 14 |
| ACA53380 | Choristoneura occidentalis cypovirus 16 |
| AHJ14791 | Inachis io cypovirus 2 |
| AZG03007 | Eyach virus |
| AZG03006 | Eyach virus |
| AAM18359 | Eyach virus |
| AAM18358 | Eyach virus |
| ACF49423-ACF49425 | Eyach virus |
| AND77148 | Eyach virus |
| QYV43093 | Eyach virus |
| NP_620280 | Eyach virus |
| QKK82923 | Fennes virus |
| QIS88057 | Fennes virus |
| AAK40249 | Fiji disease virus |
| QYV43106 | Gierle tick virus |
| QZR93734 | Jeddah tick coltivirus |
| UYL95536 | Kashgar Reovi tick virus 1 |
| YP_010086008 | Kundal virus |
| WAK76791 | Lishui pangolin virus |
| WAK76790 | Lishui pangolin virus |
| QIE06438 | Lishui pangolin virus |
| URG17203 | Lishui pangolin virus P196T/pangolin/2018 |
| AMU04173 | Mahlapitsi orthoreovirus |
| ANG56321 | Maize rough dwarf virus |
| AAO73182 | Mal de Rio Cuarto virus |
| AAL36027 | Mammalian orthoreovirus 4 Ndelle |
| ANJ21365 | Mammalian orthoreovirus 1 |
| AGG40205 | Mammalian orthoreovirus 2 |
| ADJ00316 | Mammalian orthoreovirus 3 Dearing |
| AAP45577 | Cryphonectria parasitica mycoreovirus-1 (9B21) |
| BAC98431 | Mycoreovirus 3 |
| BFG88135 | Nakatsu tick virus |
| UYL95529 | Nanning Reovi tick virus 1 |
| UYL95538 | Nanning Reovi tick virus 2 |
| WHP37825 | Nelson Bay orthoreovirus |
| BAQ19494 | Pycnonotidae orthoreovirus |
| BAA08542 | Nilaparvata lugens reovirus |
| UJQ88099 | O'hara headland virus |
| UXL90843 | Qinghe tick reovirus |
| WWV88571 | Reoviridae sp. |
| WWV88567 | Reoviridae sp. |
| WWV88564 | Reoviridae sp. |
| WWV88562 | Reoviridae sp. |
| WWV88561 | Reoviridae sp. |
| WWV88558 | Reoviridae sp. |
| WAK76796 | Reoviridae sp. |
| WAK76797 | Reoviridae sp. |
| WWV87832 | Reoviridae sp. |
| WAK76800 | Reoviridae sp. |
| WAK76803 | Reoviridae sp. |
| WAK76785 | Reoviridae sp. |
| WAK76798 | Reoviridae sp. |
| WAK76806 | Reoviridae sp. |
| WWV88207 | Reovirus sp. |
| CAC82519 | Rice black streaked dwarf virus |
| AAC36456 | Rice ragged stunt virus |
| USH09528 | Salmon River virus |
| UVC65887 | Salmon River virus |
| QVL22803 | Shelly headland virus |
| UJQ88339 | Shelly headland virus |
| UJQ88340 | Shelly headland virus |
| UJQ88341 | Shelly headland virus |
| UJQ88342 | Shelly headland virus |
| UJQ88343 | Shelly headland virus |
| AYP67545 | Shelly headland virus |
| CBH31251 | Southern rice black-streaked dwarf virus |
| YP_010839657 | Tai Forest reovirus |
| BBA54722 | Tarumizu tick virus |
| YP_010086016 | Tarumizu tick virus |
| BBA54748 | Tarumizu tick virus |
| BBK20271 | Tarumizu tick virus |
| BDB06937 | Tarumizu tick virus |
| AOM63686 | chelonian orthoreovirus |
| UYL95532 | Yanbian Reovi tick virus 1 |
| UYL95533 | Yanbian Reovi tick virus 2 |
| UYL95534 | Yanbian Reovi tick virus 3 |
| CAG9164797 | Zeboroti virus |
| UYL95530 | Zhangjiakou Reovi tick virus 1 |
| WYW04511 | Reoviridae sp. |
| WYW04510 | Reoviridae sp. |
| WZL61382 | Calla Lily Valley virus |
|  |  |
| **VP2 Gene** | |
| **Accession** | **Organism_Name** |
| ACF49426 | Eyach virus |
| ACF49427 | Eyach virus |
| ACF49428 | Eyach virus |
| AND77149 | Eyach virus |
| AYP67546 | Shelly headland virus |
| BAS02070 | Cryphonectria parasitica mycoreovirus-1 (9B21) |
| BBA54723 | Tarumizu tick virus |
| BBA54749 | Tarumizu tick virus |
| BBK20272 | Tarumizu tick virus |
| BDB06936 | Tarumizu tick virus |
| BFG88136 | Nakatsu tick virus |
| NP_620281 | Eyach virus |
| NP_690892 | Colorado tick fever virus |
| QIE06439 | Lishui pangolin virus |
| QIS88058 | Fennes virus |
| QYV43094 | Eyach virus |
| QYV43107 | Gierle tick virus |
| QZR93735 | Jeddah tick coltivirus |
| UJQ88344 | Shelly headland virus |
| UPN67770 | California hare coltivirus |
| USH09529 | Salmon River virus |
| UVC65862 | Colorado tick fever virus |
| UVC65875 | Colorado tick fever virus |
| UVC65888 | Salmon River virus |
| UXL90844 | Qinghe tick reovirus |
| WHS68119 | Colorado tick fever virus |
| WKE34862 | Colorado tick fever virus |
| WKE34869 | Colorado tick fever virus |
| WKE34874 | Colorado tick fever virus |
| WKE34884 | Colorado tick fever virus |
| WKE34901 | Colorado tick fever virus |
| WKF25357 | Colorado tick fever virus |
| WKF25359 | Colorado tick fever virus |
| WWV87811 | Reoviridae sp. |
| WWV87834 | Reoviridae sp. |
| WWV88557 | Reoviridae sp. |
| WWV88560 | Reoviridae sp. |
| WWV89314 | Riboviria sp. |
| WZL61383.1 | Calla Lily Valley virus |
| XCO66240.1 | Colorado tick fever virus |
| XKB76581 | Reovirales sp. |
| XKB76582 | Reovirales sp. |
| YP_001936005 | Mycoreovirus 1 |
| YP_009252406 | Sclerotinia sclerotiorum mycoreovirus 4 |
| YP_010086009 | Kundal virus |
| YP_010086015 | Tarumizu tick virus |
| YP_010839655 | Tai Forest reovirus |
| YP_392476 | Mycoreovirus 3 |
| NC_007154.1 | Fiji disease virus |
